# Impact of the AGE-ON Tablet Training Program on Social Isolation, Loneliness, and Attitudes Toward Technology in Older Adults: Single-Group Pre-Post Study

**DOI:** 10.2196/18398

**Published:** 2020-04-20

**Authors:** Sarah E Neil-Sztramko, Giulia Coletta, Maureen Dobbins, Sharon Marr

**Affiliations:** 1 School of Nursing McMaster University Hamilton, ON Canada; 2 School of Kinesiology McMaster University Hamilton, ON Canada; 3 Division of Geriatrics McMaster University Hamilton, ON Canada

**Keywords:** technology, older adults, tablet training, education

## Abstract

**Background:**

The internet and technology can help older adults connect with family and friends. However, many older adults face obstacles to internet and technology use, such as lack of knowledge or self-efficacy.

**Objective:**

The purpose of this study was to explore the impact of the AGE-ON tablet training program on social isolation, loneliness, and quality of life.

**Methods:**

Adults aged >60 years took part in a series of 6 weekly workshops covering the basic features of a tablet. Before and after the program, social isolation, loneliness, social support, and quality of life were assessed. In addition, data on current tablet use and attitudes toward technology use were collected. Satisfaction with the program was also assessed at the end of the study using 6 Likert scale questions.

**Results:**

The participants (N=32; mean age 76.3, SD 8.6 years) were predominantly female (n=20, 63%) and retired (n=30, 94%). The participants reported that they were highly satisfied with the program. After completing the program, no differences in social isolation, loneliness, social support, or quality of life were found. Frequency of tablet use increased and the attitudes of the participants toward technology improved.

**Conclusions:**

The AGE-ON program resulted in increased tablet use frequency and may improve comfort and attitudes toward tablet use among older adults. This program may assist older adults in overcoming obstacles to internet and technology use to better connect with family and friends; however, further work targeting older adults who are socially isolated or at risk of social isolation is needed to more fully understand whether tablet training programs are beneficial in this population.

**Trial Registration:**

ClinicalTrials.gov NCT03472729; https://clinicaltrials.gov/ct2/show/NCT03472729

## Introduction

Canada’s cohort of adults over the age of 65 years is the fastest growing segment of the population. In 2018, 17.2% of the Canadian population was aged 65 and older; this segment is expected to reach 20% by 2024 [1]. Social isolation and loneliness are growing concerns, as individuals are living longer and living alone, often far away from family members [2]. Social isolation is marked by living alone and having infrequent social contact and/or few social network ties [3]. Loneliness is a subjective emotional experience in which there is dissatisfaction with the discrepancy between desired and actual social connections [4]. Both social isolation and loneliness are associated with poor quality of life [5] and premature mortality [6].

Use of the internet and technology has been proposed as a way for older adults to connect with family and friends, thus maintaining or even enhancing social connections, reducing loneliness and isolation, and improving quality of life [7-10]. The use of the internet for interpersonal communication and information seeking is positively correlated with life satisfaction and negatively correlated with depression in older adults [11]. However, older adults have barriers to internet and technology use, such as lack of knowledge and self-efficacy and concerns about privacy [12]. Although internet use among people over the age of 65 years is steadily increasing, older adults are still less likely to use the internet than younger age groups [13]. Internet use declines with increasing age, from 60% of persons aged 65-74 years to 29% of persons aged >75 years [14].

To date, several internet-based and computer-based training programs have been reported in the literature. A 2012 meta-analysis of 5 studies suggests that computer and internet training interventions can reduce loneliness in older adults, although the included studies reported small sample sizes and had high risk of bias [15]. More recently, a systematic review of interventions targeting loneliness and social isolation found that technology and community-engaged arts may be the most effective interventions to achieve these outcomes [9].

AGE-ON is a series of workshops developed in response to this literature by the Regional Geriatric Program Central (RGPc), based in Hamilton, Ontario. The goal of these workshops is to teach older adults with limited computer knowledge how to use tablet computers to connect with friends and family and gather information related to issues of importance to them. The primary objective of this study was to evaluate the effectiveness of this program at improving the participants’ level of social isolation. The secondary objectives were to explore the impact of the program on loneliness and quality of life in older adults, the participants’ comfort with and use of the tablet, and their satisfaction with the program.

## Methods

### Study Design

This is a single-group pre-post program evaluation of the existing AGE-ON program, which is administered by the RGPc. The study was registered prior to launch (ClinicalTrials.gov; NCT03472729). Our original intent was to conduct a randomized controlled trial with a wait-list control group; however, due to feasibility within the RGPc, namely lack of staff time and availability to host a second session at each location for wait-list control participants, this was not possible, and a single-group design was used. All procedures were reviewed and approved by the Hamilton Integrated Research Ethics Board, and all participants provided informed consent.

### Participants

Eligible participants were English-speaking adults who expressed interest in the AGE-ON program. No exclusion criteria were applied. Recruitment was conducted via advertisements in local community newspapers, community postings, online postings, and social media targeting friends and caregivers of older adults from September 2018 to August 2019. All interested participants contacted the AGE-ON program coordinator at the RGPc, who provided information about the workshops and informed the participants about the research. Participation in the research component was optional and was not required to take part in the workshops. Participants did not receive compensation for their participation in the study; however, the program registration fee of CAD $40 (US $28.40) was waived for the participants.

### Study Protocol

Participants attended 2-hour education sessions weekly over 6 consecutive weeks. Each session was facilitated by 1 instructor, with support from 2-4 volunteer university student mentors. The workshops were delivered in several settings, including retirement residences and an auditorium of a local hospital. The AGE-ON program, originally named iLive iLearn Well, was developed in 2014 by the RGPc to help older adults engage with technology and decrease perceived social isolation. The educational content was divided into 5 detailed modules, with 1 education session left free for review and participant-specific questions. The first week of classes focused on learning the basic features of an iPad (eg, powering on and off, volume, locating controls) and locating the variety of available apps. In subsequent weeks, participants learned how to use specific applications, including using the internet, taking and viewing photographs, sending and receiving emails, and using basic apps (eg, the Maps app, the Clock app, and Siri). The modules were accompanied by a participant workbook that included session content, homework, and additional information to help the participants learn the material. The homework assignments corresponded with the modules; they expanded on specific concepts and skills or prepared participants for future education workshops.

### Outcome Measures

Quantitative data were collected at baseline and at the end of the 6-week program during the first and last AGE-ON sessions via paper questionnaires. Follow-up data were collected 1 month following the workshops by a telephone call with a trained research assistant (social support, attitudes toward technology, and tablet usage patterns only). The primary outcome was change in self-reported social isolation using the Duke Social Support Index (DSSI) [16]. The DSSI is an 11-item self-report scale that provides a measure of an individual’s level of social isolation. Secondary outcomes included level of loneliness, determined using the De Jong Gierveld Loneliness Scale, which has been found to be a reliable and valid assessment of emotional and social loneliness [17]; quality of life, using the validated CASP-19 questionnaire [18] that is designed specifically for older adults with a focus on overall self-perceived quality of life; social support, using the 12-item Lubben Social Network Scale [19]; and comfort in using the internet and iPad, using a variation of the Older Adults’ Computer Technology Attitudes Scale [20] that was modified to be relevant to tablet use. The feasibility of the program was also assessed by collecting participant feedback on acceptability, participant satisfaction, and intent to continue use of the iPad, using a 6-point Likert scale. Demographic data were collected at baseline, including age, gender, education, employment status, marital status, living arrangement, and racial group.

### Data Analysis

All statistical analyses were completed in SPSS version 9.4 (IBM Corporation). Baseline demographic data were summarized as mean and standard deviation or frequency and percentage where appropriate. Changes in outcome measures from baseline to end of study and 1-month follow-up were analyzed using the paired *t* test for continuous data and the chi-square test for dichotomous data.

## Results

A total of 32 participants took part in the research study over 4 offerings of the program. No participants were found to be ineligible, but the method of recruitment was not tracked. The demographic characteristics of the study participants are presented in Table 1. The mean age of the 32 participants was 76.3 years (range 64-94); the majority were female (20, 63%), white (29, 91%), and retired (30, 94%). The individuals were well educated: of the 32 participants, 6 (19%) had a college or bachelor’s degree and 11 (34%) had received a postgraduate degree or postgraduate training. Half the participants reported living with another person, such a wife, husband, partner, or children.

There were no significant differences in social isolation when measured either as a total score or as interaction or support sub-scores between baseline and end of study or at 1-month follow-up (Table 2). Moreover, no differences were found for loneliness, quality of life, or social support at 1-month follow-up. The participants’ attitudes toward technology were not significantly different after they took part in the program (+3.9, 95% CI –2.4 to 10.3; *P*=.22) but did increase 1 month later (+10.1, 95% CI 3.6-16.6; *P*=.004).

The participants’ self-reported frequency of tablet use increased from baseline to end of study; this increase was maintained at follow-up, changing from an average of several times a month to once a week (Table 3). There was also a significant increase in the number of reported uses of the tablet, from an average of 2.9 at baseline to 4.0 at end of study (*P*=.001) and at follow-up (*P*=.002). The most common tablet uses were email, seeking an answer to a specific question, internet browsing, and seeking health information. The only statistically significant difference between time points was an increase in the proportion of participants who used their tablet to seek health information (41.4% at baseline vs 62.1% at end of study, *P*=.04).

Overall, the participants were highly satisfied with the program, with 23 (71.9%) of the 32 participants finding the information useful, 22 (68.8%) indicating they would be interested in future workshops, and 25 (78%) reporting they would recommend the workshop to family and friends (Table 4). However, fewer participants reported that they intended to use (21, 66%) or had actually used (12, 38%) their iPad more because they took part in the program, and only 25 (56%) indicated that they shared the information learned with family and friends.

**Table 1:** Descriptive characteristics of the study participants at baseline (N=32).

Characteristic		Value
Age, mean (SD)		76.3 (8.6)
Gender, female, n (%)		20 (62.5)
**Education, n (%)**		
	High school diploma, GED^a^ diploma, or less	8 (25.0)
	Some college, vocational, or training school after high school graduation	5 (15.6)
	College or bachelor’s degree	6 (18.8)
	Postgraduate degree or training	11 (34.4)
	Other	2 (6.3)
**Employment status, n (%)**		
	Retired	30 (93.8)
	Part-time employment	1 (3.1)
	Other	1 (3.1)
**Marital status, n (%)**		
	Widowed	13 (40.6)
	Presently married or living with a partner	12 (37.5)
	Divorced or separated	5 (15.6)
	Never married	2 (6.3)
**Living situation, n (%)**		
	With a wife, husband, or partner	12 (37.5)
	Alone	10 (31.3)
	In a retirement home	6 (18.8)
	With children	3 (9.4)
	With someone else	1 (3.1)
**Racial group, n (%)**		
	White	29 (90.6)
	Asian	1 (3.1)
	Black or African American	1 (3.1)
	Other	1 (3.1)

^a^GED: General Educational Development.

**Table 2:** Quantitative outcomes at baseline, end of study, and follow-up.

Outcome		Baseline, mean (SD)	Change at end of study			Change at 1-month follow-up	
			Mean (95% CI)		*P* value	Mean (95% CI)	*P* value
**Social support**							
	Total score	27.6 (3.9)	+0.3 (–1.1 to 1.8)		.61	+0.6 (–1.1 to 2.2)	.49
	Interaction subscale	9.1 (1.9)	-0.03 (–0.6 to 0.6)		.91	+0.1 (–0.7 to 0.9)	.78
	Support subscale	18.5 (2.9)	+0.4 (–0.8 to 1.5)		.53	+0.4 (–0.7 to 1.6)	.43
Attitudes toward technology		13.5 (14.3)	+3.9 (–2.4 to 10.3)		.22	+10.1 (3.6 to 16.6)	.004
Loneliness		31. 9 (5.9)	+1.2 (–0.4 to 2.9)		.13	—^a^	—
Quality of life		40.4 (9.8)	+0.6 (–2.5 to 3.8)		.68	—	—
Social isolation		16.0 (5.8)	+0.1 (–1.6 to 1.9)		.87	—	—

^a^Not measured.

**Table 3:** Participant tablet usage patterns before and after the AGE-ON program.

Usage pattern		Baseline (N=32)	End of study (N=29^a^)		One-month follow-up (N=27^b^)	
			Value	*P* value	Value	*P* value
**Tablet use frequency, mean (SD)**						
	Frequency of tablet use^c^	2.9 (2.1)	4.0 (1.5)	.001	4.0 (2.0)	.002
	Number of reported tablet uses	3.1 (2.4)	4.3 (2.1)	.06	4.4 (2.2)	.01
**Tablet use type, n (%)**						
	Email	19 (65.5)	23 (79.3)	.37	21 (77.8)	.09
	Instant messaging	5 (17.2)	10 (34.5)	.19	7 (25.9)	.09
	Internet browsing	12 (41.4)	18 (62.1)	.26	21 (77.8)	.68
	Audio or video calling	4 (13.8)	8 (27.6)	.28	8 (29.6)	.51
	Reading the news	10 (34.5)	16 (55.2)	.24	9 (33.3)	.08
	Reading an e-book	4 (13.8)	5 (17.2)	.06	5 (18.5)	.08
	Answering a question	15 (51.7)	20 (69.0)	.78	21 (77.8)	.30
	Seeking health information	12 (41.4)	18 (62.1)	.04	19 (70.4)	.40
	Social media	9 (31.0)	7 (24.1)	.87	7 (25.9)	.24

^a^Three participants did not complete the baseline checklist.

^b^An additional 2 participants were missing data at follow-up.

^c^Assessed using a 6-point Likert scale: 1=never to 6=every day.

**Table 4:** Participant satisfaction with the AGE-ON program.

Survey prompt	Survey responses, n (%)		
	Strongly agree	Neutral	Strongly disagree
The information was useful.	23 (71.9)	6 (18.8)	0 (0)
I would be interested in future workshops.	22 (68.8)	7 (21.9)	0 (0)
I have shared the information I learned with family or friends.	18 (56)	9 (28)	2 (6)
I would recommend the workshop to family or friends.	25 (78)	4 (13)	0 (0)
Because of the workshop, I intend to use my iPad or tablet more.	21 (66)	7 (22)	1 (3)
Because of the workshop, I have used my iPad or tablet more.	12 (38)	7 (22)	1 (3)

## Discussion

The purpose of this study was to explore whether taking part in a real world offering of a 6-week tablet training program for older adults reduced social isolation or loneliness or improved quality of life. Overall, despite high levels of satisfaction with the program itself, no changes were observed in our primary or secondary outcomes of interest.

These findings are in contrast to a 2012 meta-analysis that found a statistically significant decrease in loneliness scores in older adults when the results of 5 studies were pooled together, with an effect size of 0.56 [15]. Within this meta-analysis, the largest effects were found in studies that included individuals who were living in nursing home facilities and those who took part in adult day-care programs [21,22]. These participants may have started with higher levels of loneliness at baseline; thus, it would be more likely to see effects of the intervention on this outcome. The Lubben Social Support Scale utilized in this study categorizes a person with a score of less than 12 as “at risk” for social isolation. Within our study, only 5 of 32 participants (16%) scored below this cutoff value at baseline, with a mean score across all participants of 16.0 (SD 5.8). Participants in the AGE-ON program were older adults who were interested in taking part in a tablet training program to learn this new skill and were not specifically identified because they were at risk for social isolation. Recruitment strategies were targeted at teaching older adults how to use tablet computers in a welcoming environment and were not targeted to lonely or socially isolated older adults. Therefore, it is possible that if this program were delivered to older adults who experience loneliness or who are at risk for social isolation, the findings with respect to this outcome would be different.

Our findings are similar to a previous study that found no difference in social isolation or self-esteem in older adults with psychiatric conditions who took part in twice-weekly internet and technology training over the course of 6 weeks [23]. These participants also reported high satisfaction with the program, and the investigators suggested that a longer training program would be needed to see meaningful improvements. Another study found no difference in social support among older adults who were assigned to learn how to use social networks or an online diary website vs a wait-list control group [24]; these participants also rated the intervention favorably. A more recent updated systematic review of 25 studies of the effects of various types of information and communication technology on social isolation, social support, social connectedness, and loneliness and depression also found inconclusive results [25]. The types of interventions included in this review were broader and included items such as landline telephone–based befriending services and mobile phone instant messaging apps; the results indicated that the consistency of the findings with respect to social isolation or loneliness is more closely related to the population included than to the intervention used.

At the end of the 6-week AGE-ON program and 1 month later, the participants’ attitudes toward technology were more favorable than at baseline; also, the participants self-reported that they used their tablets more frequently and for a wider variety of uses. Therefore, we are confident that the program was effective in helping to teach the participants how to use the iPad and that it met their learning needs. It is likely that the lack of change in social isolation and loneliness is not due to a failure of the program in teaching the participants to use the tablet properly but is rather due to the fact that using the tablet itself does not reduce social isolation or loneliness in this population. This is not surprising when we consider the types of activities for which the participants primarily reported using an iPad. The most common activities across all 3 time points were using email, finding the answer to a specific question, and seeking health information. Although email can be used to connect with others, other activities that would likely contribute more to feelings of social connectedness and reducing loneliness, such as audio or video calling, instant messaging, and social media, were reported by less than one-third of participants.

An emerging concern with respect to the use of technology is its ability to actually increase feelings of social isolation or loneliness in users. In a recent qualitative study of older adults aged ≥70 years who regularly used social media or social technology, the participants stated that while social media and technology use could certainly strengthen existing social relationships and bring depth and fun to social contacts, technology could also represent an obstacle to real human contact [26]. Encouragingly, in our study, we did not see negative changes in any of the measures of social isolation, loneliness, or social support; however, this is an important aspect that should be considered in future research.

Several methodological considerations limit the interpretation of our findings. First, as this was an evaluation of an ongoing community-based program, we were not able to randomize the participants to a control group. Second, this study included a convenience sample of highly motivated individuals who were offered free participation in a tablet training program and who mostly had access to a tablet of their own either at home or through a family member or friend. Thus, these results may be less applicable to the broader population. Finally, given the timeframe of the funding opportunity, we were only able to evaluate 4 offerings of the program in which 32 participants took part. Although a large sample would provide greater power to detect statistically significant differences, due to the consistent lack of change in any of the outcomes related to social isolation, loneliness, and social support, we do not believe a larger sample would alter our conclusions.

Overall, this study found that while older adults who took part in a tablet training program enjoyed the program and learned skills related to using a tablet computer and technology in general, participation in the program did not result in changes to social isolation, loneliness, or social support. Future work that specifically targets older adults who are socially isolated or at risk of social isolation is needed to more fully understand whether tablet training programs are beneficial in this population.

## Abbreviations

DSSI: Duke Social Support Index
RGPc: Regional Geriatric Program Central
